# Supplementary figures and images for: Can silicon applied to correct soil acidity in combination with Azospirillum brasilense inoculation improve nitrogen use efficiency in maize?
Source: PLoS One. 2020 Apr 8;15(4):e0230954. doi: 10.1371/journal.pone.0230954 (PMC7141695; doi:10.1371/journal.pone.0230954)

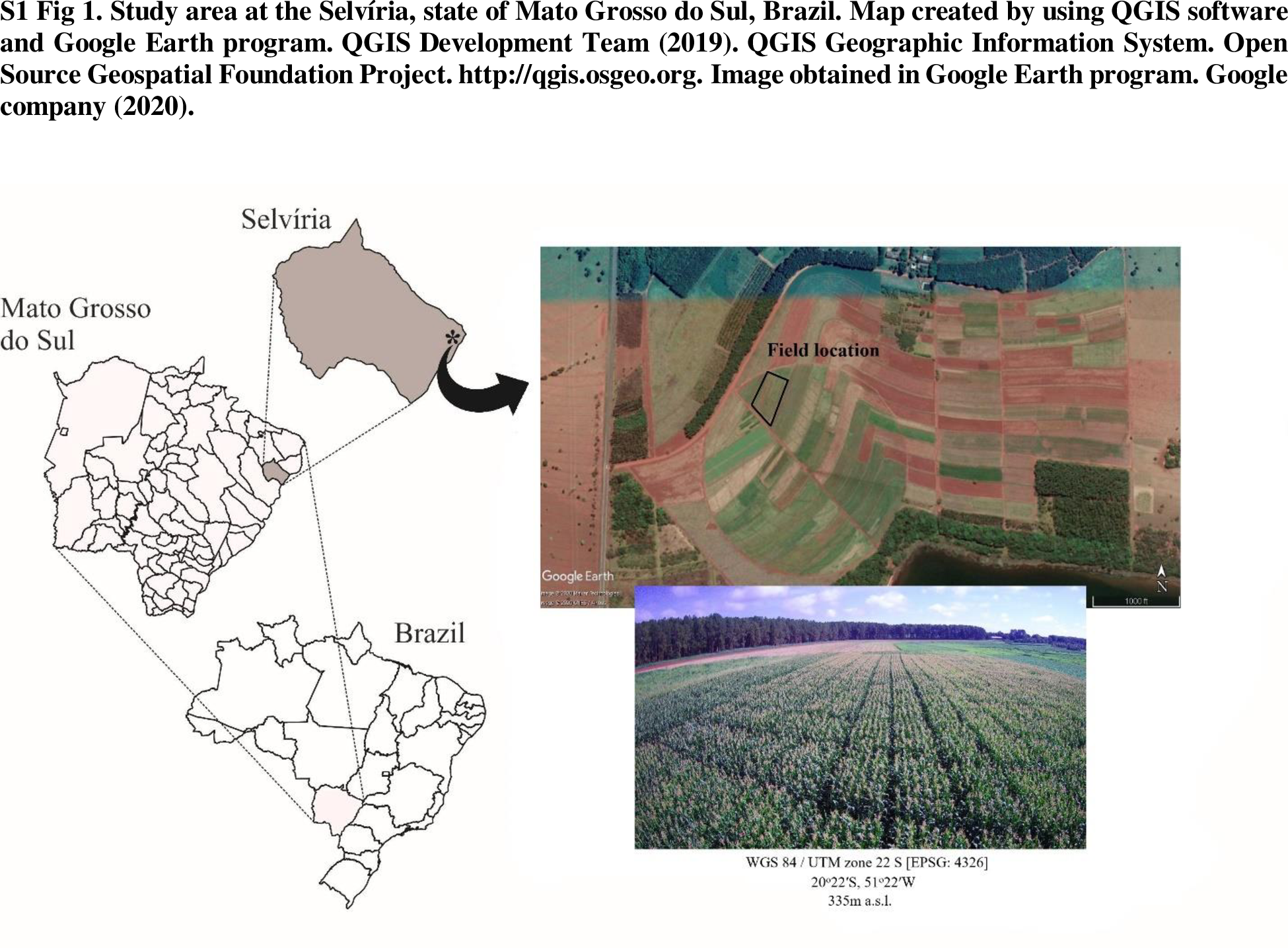

Supplement: S1 Fig — Image obtained in Google Earth program. Google company (2019). (TIF) [file pone.0230954.s001.tif]
