# Supplementary material for: Can silicon applied to correct soil acidity in combination with Azospirillum brasilense inoculation improve nitrogen use efficiency in maize?
Source: PLoS One. 2020 Apr 8;15(4):e0230954. doi: 10.1371/journal.pone.0230954 (PMC7141695; doi:10.1371/journal.pone.0230954)
Supplement: Attachment — Submitted filename: comments_24172 - Copy.docx [file pone.0230954.s003.docx]

PONE-D-19-24172

Can silicon applied to correct soil acidity in combination with *Azospirillum brasilense* inoculation improve nitrogen use in maize?

The titled manuscript was conducted to evaluate the interactive effects of Si and inoculation on maize yield and N uptake, the study provides good insights and the results will be useful in future on similar topic. However, there are few concerns which should be addressed (especially for data analysis) before the final consideration of manuscript, and the below comments should be responded properly and addressed carefully in revised draft.

- The title can be modified by replacing the term ‘nitrogen use’ with ‘nitrogen use efficiency’
- In abstract section, mostly the authors discussed about methods, and the results/conclusion part is too brief. Please describe the results more.
- If the authors have information for lime use practice by farmers in maize crop in Brazil’s maize region, please quote
- L150 – given the ratio of compound fertilizer (08-28-16, N-P2O5-K2O), the blanket dose of N must be 30 kg ha^–1^, could you please justify?
- In methods, please provide the general practice of fertilizer input rate in maize crop. And with reference to L126-128, authors are requested to add the details of fertilizer N input to the study site for at least previous crop before the maize plantation in 2015/16.
- L170 – please mentioned the plant density in ‘m^–2^’ basis. In given dimensions, it should be 6.6 plants m^–2^.
- L188 – please provide the sampling frequency and time
- L214 – please mention the statistical package name, which was employed to analyze the data? Also mention if the year effect was considered as random or fixed? Why the data was not analyzed across the years?
- L227 – please use proper sign for interaction (×) using ‘insert > symbol’ option in word program. Same is suggested for rest of the draft, and in Table 3.
- Please rectify the errors on page 15 (L315) and page 19 (L432).
- Discussion: please discuss the possibilities of factors affecting the study results due to other rotational wheat crop in the year 2016 (L127-128), what was the N rate in wheat crop? Why not the fallow period was observed between two maize crops?? Please consider this factor in discussing overall results.
- How the y-axis scale could be same for the temperature and precipitation in Fig. 1, please recheck by adding separate scale for precipitation.
- From the figure data presented, it can be assessed that most of the times the differences between the two levels of a factor (N dose, lime sources, inoculation) are negligible or null. Please discuss this aspect in the results section or in the discussion. Secondly, mention the main effects of N dose affecting the grain yield or other salient parameters. The variance between each level shows no/slight differences. Please explain!!
