# Supplementary material for: Can silicon applied to correct soil acidity in combination with Azospirillum brasilense inoculation improve nitrogen use efficiency in maize?
Source: PLoS One. 2020 Apr 8;15(4):e0230954. doi: 10.1371/journal.pone.0230954 (PMC7141695; doi:10.1371/journal.pone.0230954)
Supplement: Attachment — Submitted filename: Galindo et al_Authors response to reviewers_january.docx [file pone.0230954.s005.docx]

Dear Academic Editor,

We would like to express our gratitude for the reviewers who took the time to provide such a thorough review of our manuscript. We believe that the changes suggested have made our manuscript much more direct and much easier to follow. We have addressed all of the concerns raised and provide a point by point answer on how we handled each comment provided. Our answers will be in *italic and underlined* right after each comment.

Again, our most sincere gratitude to you and the reviewers who took time from their busy schedule to help us making this manuscript a better paper. We hope that we have answered every inquiry to your satisfaction and also hope that you will find this version of publishable quality. Should you find that further work is needed we will also gladly do it in a timely manner.

Very best,

Authors

PONE-D-19-24172R1
Can silicon applied to correct soil acidity in combination with Azospirillum brasilense inoculation improve nitrogen use efficiency in maize?
PLOS ONE

Dear Dr. Filho,

Thank you for submitting your manuscript to PLOS ONE. After careful consideration, we feel that it has merit but does not fully meet PLOS ONE’s publication criteria as it currently stands. Therefore, we invite you to submit a revised version of the manuscript that addresses the points raised during the review process.
==============================
ACADEMIC EDITOR: Reviewers raised concerns about the quality of the figures. Authors should improve the quality of the figures and upload again.

*R: The authors appreciate the editor comments* *and restructured all the figures. Thank you!*

==============================
We would appreciate receiving your revised manuscript by Mar 01 2020 11:59PM. When you are ready to submit your revision, log on to https://www.editorialmanager.com/pone/ and select the 'Submissions Needing Revision' folder to locate your manuscript file.

If you would like to make changes to your financial disclosure, please include your updated statement in your cover letter.

To enhance the reproducibility of your results, we recommend that if applicable you deposit your laboratory protocols in protocols.io, where a protocol can be assigned its own identifier (DOI) such that it can be cited independently in the future. For instructions see: http://journals.plos.org/plosone/s/submission-guidelines#loc-laboratory-protocols

Please include the following items when submitting your revised manuscript:

- A rebuttal letter that responds to each point raised by the academic editor and reviewer(s). This letter should be uploaded as separate file and labeled 'Response to Reviewers'.
- A marked-up copy of your manuscript that highlights changes made to the original version. This file should be uploaded as separate file and labeled 'Revised Manuscript with Track Changes'.
- An unmarked version of your revised paper without tracked changes. This file should be uploaded as separate file and labeled 'Manuscript'.

Please note while forming your response, if your article is accepted, you may have the opportunity to make the peer review history publicly available. The record will include editor decision letters (with reviews) and your responses to reviewer comments. If eligible, we will contact you to opt in or out.

We look forward to receiving your revised manuscript.

Kind regards,

Umair Ashraf
Academic Editor
PLOS ONE

*R:* *The authors appreciate the information. Thank you!*

Additional Editor Comments (if provided):

Authors have done with the comments/questions asked by the reviewers., however, reviewers and myself too also have concerns about the quality of figures. Authors should improve the resolution of the figures to make it clear.
[Note: HTML markup is below. Please do not edit.]

*R: The authors appreciate the editor and reviewers comments* *and restructured all the figures. Thank you!*

Reviewers' comments:

Reviewer's Responses to Questions

**Comments to the Author**

1. If the authors have adequately addressed your comments raised in a previous round of review and you feel that this manuscript is now acceptable for publication, you may indicate that here to bypass the “Comments to the Author” section, enter your conflict of interest statement in the “Confidential to Editor” section, and submit your "Accept" recommendation.

Reviewer #1: All comments have been addressed

Reviewer #2: (No Response)

Reviewer #3: All comments have been addressed

2. Is the manuscript technically sound, and do the data support the conclusions?

The manuscript must describe a technically sound piece of scientific research with data that supports the conclusions. Experiments must have been conducted rigorously, with appropriate controls, replication, and sample sizes. The conclusions must be drawn appropriately based on the data presented.

Reviewer #1: Yes

Reviewer #2: Yes

Reviewer #3: Yes

3. Has the statistical analysis been performed appropriately and rigorously?

Reviewer #1: Yes

Reviewer #2: Yes

Reviewer #3: Yes

4. Have the authors made all data underlying the findings in their manuscript fully available?

The [PLOS Data policy](http://www.plosone.org/static/policies.action#sharing) requires authors to make all data underlying the findings described in their manuscript fully available without restriction, with rare exception (please refer to the Data Availability Statement in the manuscript PDF file). The data should be provided as part of the manuscript or its supporting information, or deposited to a public repository. For example, in addition to summary statistics, the data points behind means, medians and variance measures should be available. If there are restrictions on publicly sharing data—e.g. participant privacy or use of data from a third party—those must be specified.

Reviewer #1: Yes

Reviewer #2: Yes

Reviewer #3: Yes

5. Is the manuscript presented in an intelligible fashion and written in standard English?

PLOS ONE does not copyedit accepted manuscripts, so the language in submitted articles must be clear, correct, and unambiguous. Any typographical or grammatical errors should be corrected at revision, so please note any specific errors here.

Reviewer #1: Yes

Reviewer #2: Yes

Reviewer #3: Yes

**6. Review Comments to the Author**

Please use the space provided to explain your answers to the questions above. You may also include additional comments for the author, including concerns about dual publication, research ethics, or publication ethics. (Please upload your review as an attachment if it exceeds 20,000 characters)

Reviewer #1: Although, the authors have responded well to all the comments raised by me, the quality of figures is too poor. Authors should check the Journal's guidelines regarding the minimum quality of figures

*R: We have made our best attempts to address those issues. We replot all the figures as suggested by the reviewer. We hope that we have answered every inquiry to your satisfaction and also hope that you will find this version of publishable quality. Should you find that further work is needed we will also gladly do it in a timely manner.*

*Thank you!*

Reviewer #2: The expression of the manuscript has been substantially improved after modification. The author also supplemented relevant information in the paper for my questions.

*R: The authors appreciate the reviewer comments. Thank you!*

Reviewer #3: Authors have addressed all the comments in revised version, and now the revised version looks good. However, authors didn't address the comment No. 9 during 1st review round. Please use proper symbol for interaction. Also the figures quality is even poor than the 1st submission, the minimum resolution of all figures should be 300dpi, please consider revising.
Thanks

*R: We have made our best attempts to address those issues.* *We replot all the figures as suggested by the reviewer. Also, we have changed all “x” by “×” in the interactions as suggested by the reviewer. We hope that we have answered every inquiry to your satisfaction and also hope that you will find this version of publishable quality. Should you find that further work is needed we will also gladly do it in a timely manner.*

*Thank you!*

7. PLOS authors have the option to publish the peer review history of their article ([what does this mean?](https://journals.plos.org/plosone/s/editorial-and-peer-review-process#loc-peer-review-history)). If published, this will include your full peer review and any attached files.


If you choose “no”, your identity will remain anonymous but your review may still be made public.


**Do you want your identity to be public for this peer review?** For information about this choice, including consent withdrawal, please see our [Privacy Policy](https://www.plos.org/privacy-policy).

Reviewer #1: Yes: Saddam Hussain

Reviewer #2: No

Reviewer #3: No

[NOTE: If reviewer comments were submitted as an attachment file, they will be attached to this email and accessible via the submission site. Please log into your account, locate the manuscript record, and check for the action link "View Attachments". If this link does not appear, there are no attachment files to be viewed.]

While revising your submission, please upload your figure files to the Preflight Analysis and Conversion Engine (PACE) digital diagnostic tool, <https://pacev2.apexcovantage.com/>. PACE helps ensure that figures meet PLOS requirements. To use PACE, you must first register as a user. Registration is free. Then, login and navigate to the UPLOAD tab, where you will find detailed instructions on how to use the tool. If you encounter any issues or have any questions when using PACE, please email us at [figures@plos.org](mailto:figures@plos.org). Please note that Supporting Information files do not need this step.

*R:* *The authors appreciate the information and attend this request. Thank you!*
